# A Double Dwell High Sensitivity GPS Acquisition Scheme Using Binarized Convolution Neural Network

**DOI:** 10.3390/s18051482

**Published:** 2018-05-09

**Authors:** Zhen Wang, Yuan Zhuang, Jun Yang, Hengfeng Zhang, Wei Dong, Min Wang, Luchi Hua, Bo Liu, Longxing Shi

**Affiliations:** National ASIC System Engineering Research Center, Southeast University, 2 Sipailou, Nanjing 210096, China; 230119228@seu.edu.cn (Z.W.); dragon@seu.edu.cn (J.Y.); zhhf@seu.edu.cn (H.Z.); dongwei_seu@seu.edu.cn (W.D.); wangmin93@seu.edu.cn (M.W.); hualuchi@gmail.com (L.H.); liubo_cnasic@seu.edu.cn (B.L.); lxshi@seu.edu.cn (L.S.)

**Keywords:** GPS acquisition, binarized convolution neural network, high sensitivity, double dwell

## Abstract

Conventional GPS acquisition methods, such as Max selection and threshold crossing (MAX/TC), estimate GPS code/Doppler by its correlation peak. Different from MAX/TC, a multi-layer binarized convolution neural network (BCNN) is proposed to recognize the GPS acquisition correlation envelope in this article. The proposed method is a double dwell acquisition in which a short integration is adopted in the first dwell and a long integration is applied in the second one. To reduce the search space for parameters, BCNN detects the possible envelope which contains the auto-correlation peak in the first dwell to compress the initial search space to 1/1023. Although there is a long integration in the second dwell, the acquisition computation overhead is still low due to the compressed search space. Comprehensively, the total computation overhead of the proposed method is only 1/5 of conventional ones. Experiments show that the proposed double dwell/correlation envelope identification (DD/CEI) neural network achieves 2 dB improvement when compared with the MAX/TC under the same specification.

## 1. Introduction

GPS signal deteriorates greatly in urban environments, and the received signal power often degrades to −143 dBm or lower, resulting in acquisition difficulties. As the mostly used acquisition method, the MAX/TC has been thoroughly studied to balance false alarm probability and detection probability [1,2,3]. However, increasing the detection probability for weak signals in GPS acquisition is still an unsolved problem. Currently, there are two ways to improve the detection probability for weak GPS signals:

- Enhancing the quality of correlation (often characterized by signal–noise-ratio) by extending the signal integration time;
- Optimizing the acquisition decision strategy by advanced techniques.

However, the performance of the first method is not good enough to acquire weak GPS signals, since the signal integration time is limited by the transition of modulated navigation data. Conventional studies of high sensitivity acquisition focus on improving the coherent integration, in which the coherent integration time has been extended to 10 ms by using large and complex computation operations [4,5,6,7]. Due to the large hypothesis parameter space existing in the cold-start environment, the computational overhead of current algorithms is fairly large, which cause them to be impractical for civilian receivers.

Lacking an efficient processing technique, the correlation envelope feature has been ignored in GPS acquisition for a long time. In recent years, convolution neural network (CNN) has attracted attention for its remarkable performance in identification and classification [8]. Motivated by its great performance, correlation envelope identification (CEI) with CNN has been introduced to compress the GPS acquisition hypothesis parameter space by identifying the envelope in a low-quality correlation. With the narrowed hypothesis parameter space, a long integration can be adopted in the follow-up acquisition to achieve a high-quality correlation. Considering the demand of huge multiplications in traditional CNN, binarized convolution neural network (BCNN) [9] is used to reduce the number of multiplications in the proposed algorithm. The BCNN weights are only +1/−1 in the network training. Moreover, the fully connected layers, which require huge computation, are replaced. It further simplifies the network complexity and reduces the computation overhead. To the best of our knowledge, this article introduces CNN to global navigation satellite system (GNSS) signal acquisition for the first time, and no other researchers have discussed the CNN-based GNSS acquisition in the past years.

The main contributions of this article are as follows:

- CNN is firstly introduced in GPS correlation envelop identification. Motivated by image recognition, the GPS acquisition correlation envelop can be detected with CNN.
- The neural network is optimized to binary CNN to reduce the computation overhead. Considering the huge calculations introduced by CNN, a binary CNN whose weights are constrained by +1/−1 is used in this article.
- The performance of the proposed algorithm is validated by the field test, and it shows that the acquisition sensitivity is improved by 2 dB under the same specification. On the other hand, in the case of the same acquisition sensitivity, the computation overhead of the proposed algorithm is only around 1/5 of conventional ones because the long integration only exists in narrowed parameter space.

The rest of this article is organized as follows. Section 2.1 states the problem which exists in conventional acquisition methods. Section 2.2 elaborates the proposed double dwell acquisition scheme. Section 2.3 presents the correlation envelope identification decision strategy based on BCNN. The test results are shown in Section 3 and this article is concluded in Section 4. 

## 2. DD/CEI GPS Acquisition Scheme

### 2.1. MAX/TC Acquisition Method

GPS is a code division multiple access (CDMA) system in which signals are modulated by binary phase shift key (BPSK). Therefore, GPS signal acquisition is the detection and estimation of code phase and Doppler frequency based on the cross-ambiguity function (CAF) [10], which is expressed as the correlation between the incoming signal and the locally generated signal.

(1) $$R(\tilde{\tau },{\tilde{f}}_{d})=|\sum_{n=0}^{N-1}c(nT_{s}-\tilde{\tau })\mathrm{sinc}({\tilde{f}}_{d}T_{s})e^{-j2\pi {\tilde{f}}_{d}nT_{s}}+w(n)|$$

where *N* is the integration time, $T_{S}$ is the code period of the signal, $c(nT_{S}-\tilde{\tau })$ is the local replica of the C/A code, $\tilde{\tau }$ and ${\tilde{f}}_{d}$ are the code delay and the Doppler shift included in the hypothesis parameter space, and *w(n)* is the additive white Gaussian noise (AWGN).

**Figure 1:**
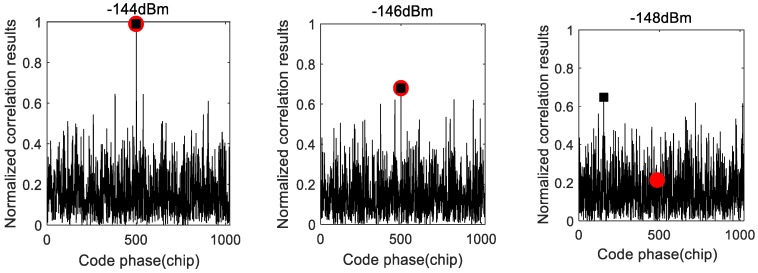
Normalized correlation results in different signal powers.

The MAX/TC acquisition method simply selects the largest correlation peak and compares it with a preset threshold. The acquisition correlations with 2 ms coherent integration for weak GPS signals are shown in Figure 1. In Figure 1, the round point marks the correlation generated by the correct parameters, while the tetragon point marks the largest correlation. The correlation peak occurs in the correct code phase when the signal power is strong. However, the correlation peak occurs in an incorrect code phase in the case of weak signals. Therefore, the MAX/TC acquisition method based on the correlation peak is impractical for weak GPS signals.

Although it is a significant method to extend the integration time to promote the performance of GPS acquisition, a long integration time with a large hypothesis parameter space will introduce high computation complexity and overhead. Therefore, apart from extending the coherent integration time, compressing the hypothesis parameter space is the inevitable choice to further improve the performance of GPS acquisition.

### 2.2. Double Dwell Acquisition Scheme

**Figure 2:**
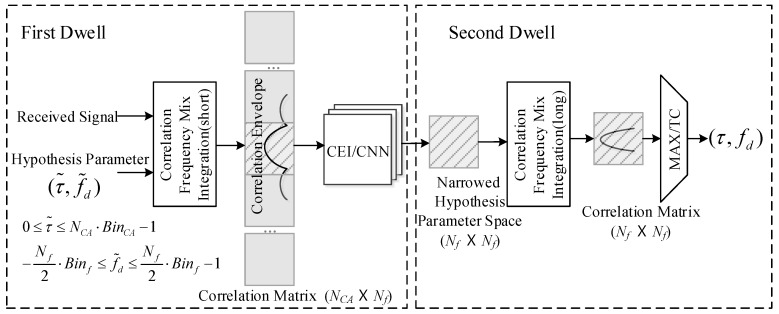
Double dwell acquisition architecture.

As shown in Figure 2, the double dwell acquisition scheme includes two steps:

1. Recognizing the most possible envelope around the correlation peak by using correlation envelope identify network (CEI) and giving the most possible parameter space;
2. Detecting the signal in the most narrowed parameter space filtered by step 1.

In the first dwell, the received signal is correlated with all the possible parameters to generate the correlation matrix, whose size is the product of code bins $N_{CA}$ and frequency bins $N_{f}$. Then, the matrix is processed by the CEI neural network and the $N_{CA\;}/N_{f}$ possibilities are output. Finally, the most likely parameter block including the right parameters is located and the possible parameter space is significantly reduced.

In the second dwell, a long coherent integration is generated by correlating between the preferred hypothesis parameters and the received signal. The most possible parameters are detected by the MAX/TC method based on the results of the high-quality correlation. 

**Figure 3:**
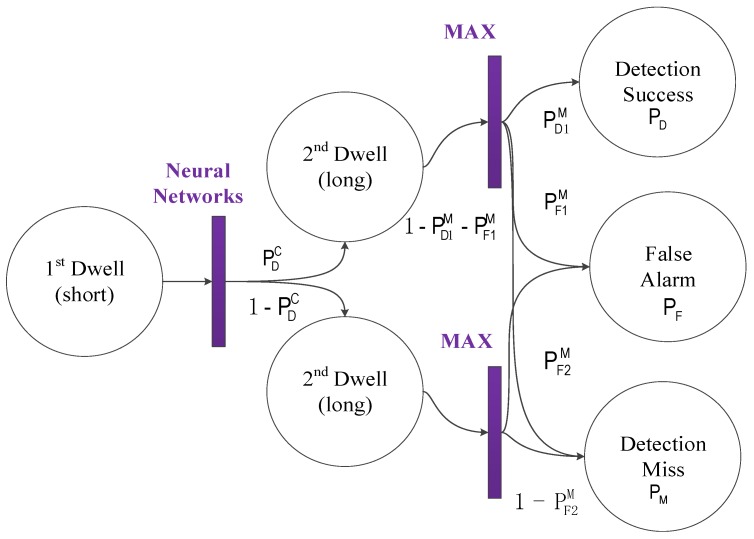
Double dwell/correlation envelope identification (DD/CEI) conditional probability graph.

The proposed acquisition method and its conditional probability are described in Figure 3. The probability $P_{D}^{C}$ is the recognition probability that the neural network recognizes the correlation envelope. If the correct envelope is recognized, there are three cases in the second dwell: (1) the real code/Doppler is detected, and its probability is defined as detection probability in the second dwell, $P_{D1}^{M}$; (2) the fake code/Doppler is detected, and its probability is defined as false alarm probability in the second dwell, $P_{P1}^{M}$; (3) misses: that is, the correlation peak does not exceed the threshold, and its probability is $1-P_{D1}^{M}-P_{P1}^{M}$. If the neural network has recognized the false envelope, there are two cases in the second dwell: (1) the fake code/Doppler is detected, and its probability is defined as false alarm probability in the second dwell, $P_{F2}^{M}$; (2) misses: that is, the correlation peak does not exceed the threshold, and its probability is $1-P_{F2}^{M}$.

From the overall acquisition perspective, the detection probability $\;P_{D}$, the false alarm probability $\;P_{F}$, and the missed detection probability $P_{M}$ are presented as follows.

(2) $$P_{D}=P_{D}^{C}\times P_{D1}^{M}$$

(3) $$P_{F}=P_{D}^{C}\times P_{F1}^{M}+(1-P_{D}^{C})\times P_{F2}^{M}$$

(4) $$P_{M}=P_{D}^{C}\times (1-P_{D1}^{M}-P_{F1}^{M})+(1-P_{D}^{C})\times (1-P_{D2}^{M})$$

Equations (2)–(4) show that the acquisition performance of the proposed algorithm is dominated by the performance of its second dwell if the recognition accuracy $P_{D}^{C}$ is high enough. Obviously, the second dwell acquisition has a better performance due to a smaller hypothesis parameter space and the results of the high-quality correlation. The overall computation overhead of the proposed method is dominated by its first dwell; and, therefore, promoting the recognition probability and decreasing computation overhead in its first dwell are both efficient methods to improve the acquisition performance. 

### 2.3. Correlation Envelope Identify Network

**Figure 4:**
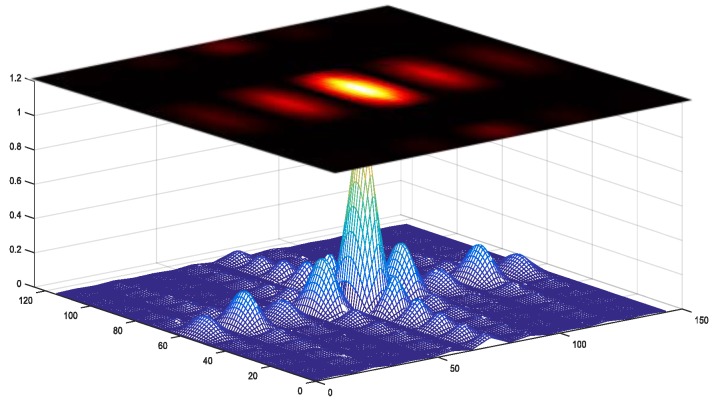
Cross-ambiguity function (CAF) envelope feature.

GPS acquisition is a two-dimensional code/Doppler correlation, and the correlation can be regarded as a grayscale “image” whose brightness represents its correlation value. When local code and Doppler are aligned with the satellite signal, there is a peak in the correlation and there is a bright block in the “image”. However, due to the weak signal, the correlation peak is not obvious, and the block is not bright enough. Conventional acquisition decision strategies only recognize the correlation peak and ignore the important envelope characteristic around the correlation peak as shown in Figure 4. In this article, the aim of the proposed neural network decision strategy is to recognize the envelope (i.e., bright block) in the “image”.

**Figure 5:**
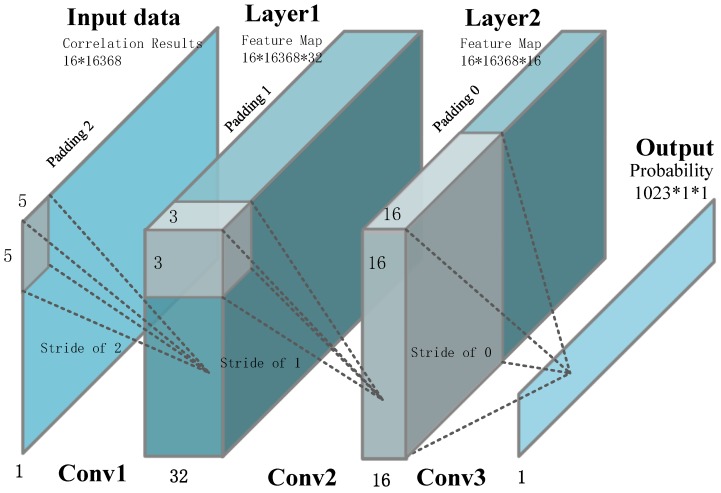
Envelope recognition by BCNN.

The input data of the proposed network is the result of the short integration correlation within the whole hypothesis parameter space, whose size is $N_{CA}\cdot N_{f}$. Considering the complexity and graininess of the CEI neural network, correlation generated with $\;N_{f}\cdot N_{f}$ parameters is regarded as a basic block, the CAF envelope is deemed to exist within one of the basic blocks. Thus, $N_{CA}/N_{f}$ output ports are included in our network, and each output port represents the probability that the CAF envelope exists within the corresponding basic block. The structure of the envelope identification neural network is demonstrated in Figure 5. The convolution layer extracts data features by using the convolution operation between input data and middle layer data. In this article, we use three convolutional layers to extract the correlation peak due to the CNN powerful noise filtering capability, whose number is the balance of precision and computation overhead. We also speed up the training convergence by batch normalization layers in the first and second convolutional layers. It is noteworthy that there is no fully connected layer in our network: the reason for this we will discuss in the next section. The detailed design parameters are described in Table 1. The stride controls how the filter convolves around the input volume, and the stride is set in a way to ensure that the output volume is an integer and not a fraction. The size and number of convolution kernel are set in a way to ensure that the network obtains a high precision in the practical test. Finally, padding is the added data around the feature map to control the size of the output.

#### 2.3.1. The Binarized Convolutional Layer

CNN has shown significant performance improvements in several applications including characteristics identification and computer vision. However, CNN-based recognitions require large amounts of memory and computational power. Therefore, they are unsuitable for smaller devices such as cell phones and embedded electronics [11]. BCNN is a network that effectively reduces the computational complexity and memory overhead. During the forward pass, BCNN drastically replaces most arithmetic calculations with bit-wise operations, which substantially improves the power-efficiency [9]. During the training, the weight is binarized to +1 or −1 for the forward pass and the calculation of gradient during the backpropagation. When updating the weight, the calculation uses the floating-point format weight. After the update, the weight will be constrained to the range of [−1, +1] by clipping. As shown in Equation (5), most calculation of the traditional CNN inference is matrix multiplication, which causes huge computation overhead. With +1/−1 weights, BCNN converts the multiplication into the XOR operation to reduce the computation overhead.

(5) $$\left[\begin{array}{ccc} W_{00} & \cdots & W_{0n}\\ \vdots & \ddots & \vdots \\ W_{m0} & \cdots & W_{mn} \end{array}\right]\times \left[x_{0},\;x_{1},\ldots ,x_{n}\right]\overset{\begin{array}{c} w^{b}=Sign\left(w\right)\\ =\{\begin{array}{c} +1,\;\mathrm{if}\;x\geq 0\\ -1,\;\mathrm{otherwise} \end{array} \end{array}}{\to }\left[\sum_{i=0}^{n}\pm x_{i}\right]$$

#### 2.3.2. The Batch Normalization Layer

During each stochastic gradient descent (SGD), the corresponding activation is normalized by the mini-batch. Therefore, the mini-batch data has a mean value of 0 and a variance of 1, which is called batch normalization. In deep networks, internal covariant shift (ICS) makes training slow and complex. Batch normalization takes a step towards reducing the ICS, and dramatically accelerates the training of deep neural networks. It accomplishes this goal via a normalization step that fixes the mean values and variances of layer inputs. Batch normalization also has a beneficial effect on the gradient flow through the network, by reducing the dependence of gradients on the scale of the parameters or of their initial values [12]. Furthermore, batch normalization can reduce the requirement of dropout operations. The batch normalization’s formula is shown below (Equation (6)). Here, *β* is the bias constant to be trained and the initial value is 0; *ε* is a very small value used to prevent the denominator from zero, and the initial value is 0.0001; *x* is the current batch normalization layer’s input, and *y* is the output. During the training, *μ* and *σ*^2^ are defined to be the mean and variance of the current input mini-batch, and during the inferring, they are replaced with average statistics over the training data.

(6) $$y=\frac{x-\mu }{\sqrt{\sigma^{2}+\varepsilon }}+\beta$$

#### 2.3.3. The Rectified Linear Unit and Fully Connected Layer

We refer to neurons with this nonlinearity as rectified linear units (ReLUs). The rectifier function is rectifier (*x*) = max (0, *x*). The activation function allows a network to easily obtain sparse representations [13]. Usually, a typical CNN network contains a few fully connected layers. Compared with the convolutional layer, the weight of the fully connected layer is not shared. The parameter size grows with the feature map size in a square relationship. In the problem of correlation envelope identification, the size of the feature map is one hundred thousand orders of magnitude, which will lead to huge memory overhead. In this work, we directly use the convolution layer instead of the fully connected layer as the output layer because the output probability is equivalent to the relative value of the feature vector in envelope identification, which significantly reduces the weight size and computation complexity.

#### 2.3.4. The Training Process

In this work, 20,000 randomly generated correlation results are used as input data for training, and 8000 correlation results are used as the test dataset. This work uses the SGD to update the network weight. During the forward pass and the calculation of gradient, the weight is binarized to +1 or −1, while the weight is updated in floating point form. The weights are initialized using the Glorot weight initialization method, which is proposed in work [14]. The pseudo code of the training process is shown below.

**Algorithm 1.** Pseudocode of the training process.**Input:** Training period p; **Output:** Network’s test accuracy; network’s test loss 1: Let dataset_training_ denote the signal data generated for training; 2: Let dataset_test_ denote the signal data generated for test;3: Let W_real_ denote the network’s weight which initialized randomly; 4: **for**
*i* = 1 to p **do**     W_binary_ = Binarized (W_real_);     loss = ForwardProcess (dataset_training_, W_binary_);     grad = BackforwardProcess (loss, W_real_);     W_new_real_ = UpdateWeight (W_real_, grad);     W_binary_ = ClipWhileExceed (W_new_real_);5: W_binary_ = Binarized (W_real_);6: [accuracy, loss] = TestForward (W_binary_, dataset_test_);7:   **return** accuracy and loss

To decrease the computation overhead in the first dwell, 2 ms coherent integration is adopted for the envelope recognition. In order to make the trained neural network suitable for different GPS signal scenes, the training set in our study is GPS signals with random power, Doppler frequency, and code phase.

**Figure 6:**
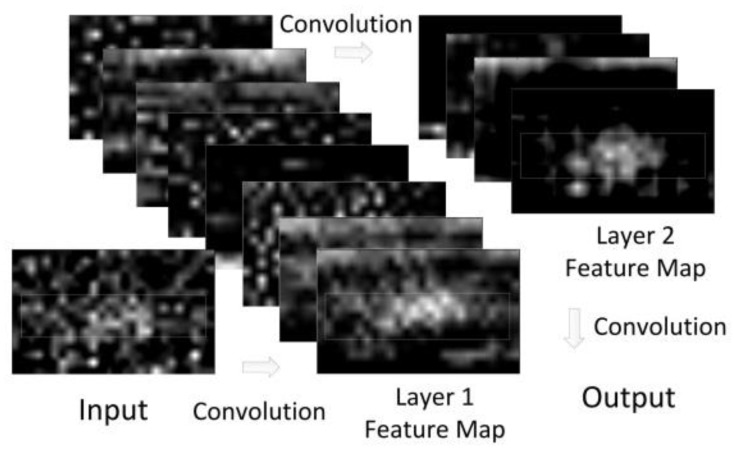
An example of the feature map in CEI network.

With the trained weights, a set of correlation results of −146 dBm GPS signal with 2 ms coherent integration is inferenced with the envelope identification and its feature map is shown in Figure 6. Obviously, the input correlation data is a low-quality data set, and its regularity cannot be recognized. With the noise reduction of our proposed network, the local feature occurs in its last layer feature map, which reveals the essential reason that the proposed network can recognize the envelope from low-quality correlation results.

## 3. Performance Validation

Several field tests were conducted to evaluate the performance of the proposed CEI neural network and its acquisition method, and one of them is selected in this article for illustration purposes. The parameters of correlation and signal model are presented in Table 2. Considering the environment of weak signals, the code bin is 1/16 chip for high sensitivity GPS acquisition. In the first dwell, the Doppler bin is 500 Hz and the coherent integration time is 2 ms for envelope identification. In the second dwell, the Doppler bin is narrowed to 100 Hz and the coherent integration time is 10 ms for acquisition. The length of GPS navigation data is 20 ms. When the coherent integration time is no more than 10 ms, navigation data transition has a small influence on the envelope characteristics of GPS signals. This research uses the coherent integration time of 2 ms and 10 ms to avoid the effect of bit jump on the acquisition results. Therefore, navigation data transition does not affect our proposed acquisition algorithm. The CEI neural network was trained by Adam with the batch size of 32 examples whose data was from the signal model. The learning rate was initialized at 0.001 and decayed every epoch where decay rate was 0.98. We trained the network for roughly 30 epochs through the training set, which took about one day on one NVIDIA Tesla K80.

The training data was generated uniformly, with its signal power typically ranging from −143 dBm to −148 dBm. The accuracy of training is 94.17%. To check the performance in the real environment, the proposed method is validated by a field test in which the signal data was collected from Spirent GPS Constellation Simulator. With 2 ms integration in the first dwell, the recognition accuracy of the proposed CEI BCNN is shown in Table 3. It shows that the recognition accuracy is high enough for the second dwell, which is 98.7%@−143 dBm and 82.4%@−148 dBm. It also can be seen that the network maintains a certain accuracy under different noises, which means the network has good generalization ability.

**Figure 7:**
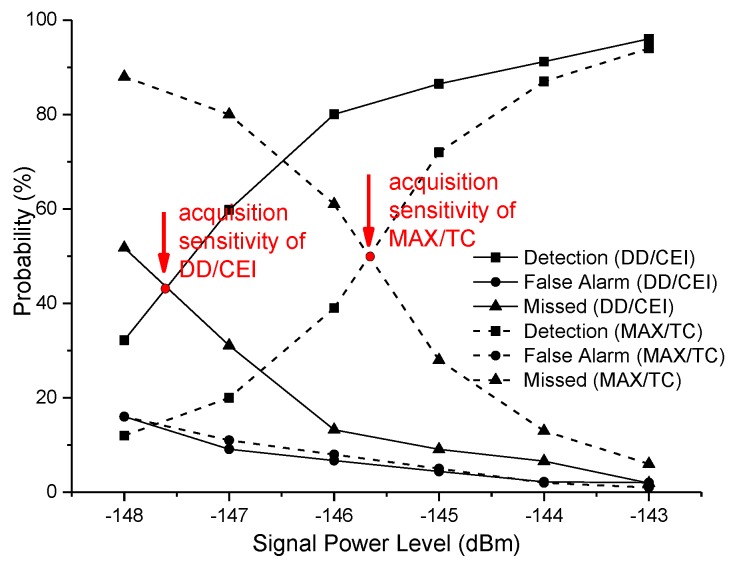
Comparison with Max selection/threshold crossing (MAX/TC).

For simplicity, the MAX/TC acquisition method was adopted in the second dwell, whose coherent integration was 10 ms. Since the conventional double dwell acquisition method uses the second dwell to verify the first dwell acquisition result, it leads to a low false alarm rate with weak signals. Thus, the conventional MAX/TC acquisition method was validated simultaneously by the same experimental environment, which adopts the 10 ms coherent integration time. Results of both methods are shown in Figure 7.

In the test, the proposed DD/CEI acquisition method adopted the same constant false alarm threshold criterion as the conventional method. Thus, the false alarm probability of this method was similar to the conventional one. When the GPS satellite signal power was −143 dBm, the detection probability of this method and the traditional method were both close to 100%. When the GPS satellite signal power was −146 dBm, the detection probability of the proposed method was 82%, which was double of the traditional method. Without loss of generality, the definition of acquisition sensitivity was the satellite power level in which its detection probability was equal to missed detection probability, that was the curve cross point of detection probability vs. missed detection probability. Therefore, the DD/CEI scheme at the condition of 2 ms integration achieved −147.6 dBm which was 2 dB improvement when compared to the MAX/TC acquisition method.

Benefit from the deep optimization on the proposed DD/CEI, the computation overhead brought by CEI neural network is from the addition of binarized convolution. In this network, the current feature map value is from the convolution of last feature map. Thus, the addition number of each feature map is the multiplication of feature map size, convolution kernel size, and the convolution kernel number. The addition number of our network is given as follows.

(7) $$S_{add}=\sum_{k=1}^{3}KS_{k}^{}{}^{2}\cdot KN_{k}^{}\cdot FN_{k}\cdot N_{f}\cdot N_{CA}$$

where $KS_{k}{}^{2}$ is the *k*th convolution kernel size, $KN_{k}$ is the *k*th convolution kernel number, $FN_{k}$ is value number of the *k*th feature map, and $S_{add}$ is about $5k\cdot N_{f}\cdot N_{CA}$.

In the traditional GPS acquisition, its computation overhead is from the integration of signal, in which the multiplication is replaced XOR. Therefore, the computation overhead of integration is the addition and the computation overhead of the coherent integration is expressed as follows:

(8) $$S_{add}=F_{s}\cdot T_{int}\cdot N_{CA}\cdot N_{f}$$

where *F_s_* is the sampling rate and $T_{int}$ is the integration time, and $S_{add}$ is $1.6M\cdot N_{f}\cdot N_{CA}$ for typical weak signal acquisition. Therefore, the computation overhead of integration in the traditional GPS acquisition is far more than the proposed CEI neural network.

**Figure 8:**
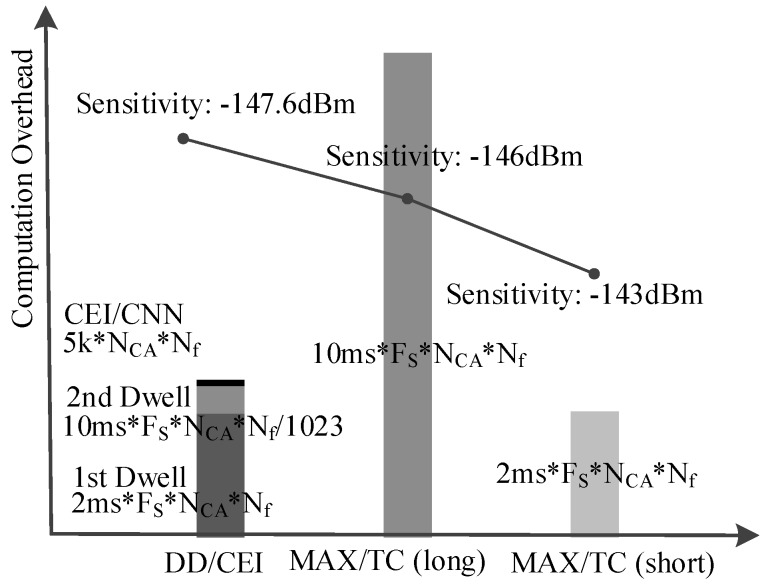
Computation overhead.

Moreover, the computation overhead of the second dwell signal integration can be ignored when compared to its first dwell as the second dwell parameter number is only 1/1023 of the first dwell. Therefore, the total computation overhead of the proposed method is mainly decided by the first dwell, which is only 1/5 of conventional MAX/TC method as shown in Figure 8.

Predictably, the proposed method will achieve a better acquisition performance if a longer integration is used for its second dwell, which will not significantly increase the computation overhead due to its small hypothesis parameter space.

## 4. Conclusions

This article proposed a novel DD/CEI neural network for high sensitivity GPS acquisition to reduce the acquisition computation overhead and promote the acquisition sensitivity. The hypothesis parameter space was significantly narrowed by the proposed neural network in the first dwell. Then, the long integration and conventional MAX/TC decision strategy were applied to the narrowed hypothesis space in its second dwell to improve the acquisition accuracy. Since the short integration was conducted on the initial hypothesis space, the computation overhead was significantly decreased. Moreover, the extra computation overhead brought by neural network was greatly reduced by weight binarization and structure optimization. Finally, field tests were conducted to evaluate the performance of the proposed algorithm. Experiment results showed that the proposed method could promote the acquisition sensitivity by 2 dB when compared with the MAX/TC under the same specification, and the computation overhead of the proposed algorithm was only around 1/5 of the MAX/TC under the same sensitivity.

## Figures and Tables

**Table 1:** Envelope recognition binarized convolution neural network (BCNN) parameters.

Name	Layer	Stride	Convolution Kernel Number	Padding	Convolution Kernel Size
Convolution Layer	1	1	32	2	5 × 5
	2	1	16	1	3 × 3
	3	16	1	0	16 × 16
Batch Normalization	Layer	Epsilon	Gamma	Beta	
	1	0.0004	1	0	
	2	0.0004	1	0	
Relu	Layer	Formal			
	1	Relu(*x*) = max(*x*,0)			
	2				

**Table 2:** Signal and correlation parameters.

Parameter	Value
Sample Frequency (MHz)	16.368
Noise Bandwidth (MHz)	4.092
Doppler Bin (Hz)	500/100
Frequency Channel (*N_f_*)	16
Code Bin (Chip)	1/16
Code Channel (*N_CA_*)	16,368

**Table 3:** Performance of BCNN decision policy.

Signal Power (dBm)	P^C^_D_ (%)
−143	98.7
−144	97.3
−145	96.0
−146	94.2
−147	89.5
−148	82.4
